# Impact of MEFV genotype in Caucasian children with periodic fever

**DOI:** 10.1186/1546-0096-9-S1-P302

**Published:** 2011-09-14

**Authors:** Silvia Federici, G Calcagno, Martina Finetti, Romina Gallizzi, Antonella Meini, A Vitale, F Caroli, M Cattalini, Roberta Caorsi, Francesco Zulian, Alberto Tomasini, A Insalaco, J Frenkel, Mariapia Sormani, M Baldi, Isabella Ceccherini, Alberto Martini, Marco Gattorno

**Affiliations:** 1 Gaslini Institute, Genova, Italy; 2 Sezione di Reumatologia Pediatrica, AOU „G. Martino„, Messina, Italy; 3 Divisione di Immunologia e Reumatologia Pediatrica,università di Messina, Messina, Italy; 4 Dipartimento di Pediatria, Unità di Immunologia e Reumatologia Pediatrica, Spedali Civili E University Of Brescia, Italy; 5 Laboratorio di Genetica Molecolare,Gaslini Institute, Genova, Italy; 6 Dipartimento A.I. di Pediatria,University of Padua, Padova, Italy; 7 IRCCS Burlo Garofolo, Dipartimento di Pediatria, University of Trieste, Trieste, Italy; 8 Divisione di Reumatologia, Ospedale Pediatrico Bambino Gesù, IRCCS, Roma, Italy; 9 Department of Pediatrics,Wilhelmina Children's Hospital,University Medical Center, Utrecht, The Netherlands; 10 Unità di Biostatistica, DISSAL, University of Genoa, Genova, Italy

## Introduction

Despite FMF is considered an autosomal recessive disease caused by mutations of *MEFV*, one third of patients carries one mutation only.

## Aim

To analyze the actual impact of MEFV mutations in children with periodic fever.

## Methods

113 caucasian patients carrying MEFV mutations (46 with mutations in two alleles, 67 heterozygous) and 205 genetically negative patients for MEFV, TNFSF1A and MEFV (70% with a PFAPA phenotype) were analyzed. The following groups were considerd: patients with: i) 2 high penetrance mutations (M694V, M694I, M680I), ii) 1 high, 1 low penetrance mutation, iii) 2 low penetrance mutations, iv) 1 high penetrance mutation, v) one low penetrance mutation, vi) genetically negative.

## Results

**Figure 1:**
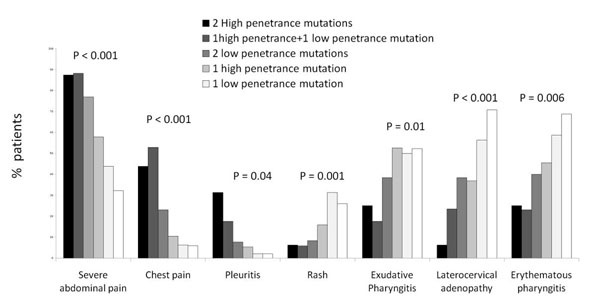
Prevalence of the clincial manifestations associated to fever attacks in patients with different MEFV genotypes (see text) and in patients with periodic fever negative for mutations of MEFV, MVK and TNFSRF1A genes. P values were assessed by a Chi-square test for trend.

Patients with two mutations displayed a higher prevalence of chest pain (p = 0.001), pleurisy (p = 0.003) and severe abdominal pain (p = 0.002) in respect to heterozygous patients, which clinical phenotype was more similar to that presented by genetically negative patients, with an higher prevalence of erythematous (p = 0.01) and exudative ( p = 0.009) pharyngitis, enlarged cervical lymph nodes (p = 0.002). The frequency of “FMF-like symptoms” decreases from patients carrying two high penetrance mutations towards patients with a single low penetrance mutation with a specular increase of “PFAPA-like symptoms” (Figure 1).

## Conclusions

The present study shows a dosage effect of MEFV mutations not consistent with a pure autosomal recessive disorder. A dominant negative or gain of function effects or variants of still unidentified modifier genes may influence the presence of a FMF phenotype in heterozygous patients.

